# Melittin restores proteasome function in an animal model of ALS

**DOI:** 10.1186/1742-2094-8-69

**Published:** 2011-06-20

**Authors:** Eun Jin Yang, Seon Hwy Kim, Sun Choel Yang, Sang Min Lee, Sun-Mi Choi

**Affiliations:** 1 Department of Standard Research, Korea Institute of Oriental Medicine, 483 Expo-ro, Yuseong-gu, Daejeon, 305-811, Korea; 2 Department of Instrument Development, Korea Basic Science Institute, 113, Gwahag-ro, Yuseong-gu, Daejeon, 305-333, Korea

**Keywords:** hSOD1^G93A^, Melittin, Amyotrophic lateral sclerosis (ALS), α-synuclein

## Abstract

Amyotrophic lateral sclerosis (ALS) is a paralyzing disorder characterized by the progressive degeneration and death of motor neurons and occurs both as a sporadic and familial disease. Mutant SOD1 (mtSOD1) in motor neurons induces vulnerability to the disease through protein misfolding, mitochondrial dysfunction, oxidative damage, cytoskeletal abnormalities, defective axonal transport- and growth factor signaling, excitotoxicity, and neuro-inflammation.

Melittin is a 26 amino acid protein and is one of the components of bee venom which is used in traditional Chinese medicine to inhibit of cancer cell proliferation and is known to have anti-inflammatory and anti-arthritic effects.

The purpose of the present study was to determine if melittin could suppress motor neuron loss and protein misfolding in the hSOD1^G93A ^mouse, which is commonly used as a model for inherited ALS. Meltittin was injected at the 'ZuSanLi' (ST36) acupuncture point in the hSOD1^G93A ^animal model. Melittin-treated animals showed a decrease in the number of microglia and in the expression level of phospho-p38 in the spinal cord and brainstem. Interestingly, melittin treatment in symptomatic ALS animals improved motor function and reduced the level of neuron death in the spinal cord when compared to the control group. Furthermore, we found increased of α-synuclein modifications, such as phosphorylation or nitration, in both the brainstem and spinal cord in hSOD1^G93A ^mice. However, melittin treatment reduced α-synuclein misfolding and restored the proteasomal activity in the brainstem and spinal cord of symptomatic hSOD1^G93A ^transgenic mice.

Our research suggests a potential functional link between melittin and the inhibition of neuroinflammation in an ALS animal model.

## Background

Amyotrophic lateral sclerosis (ALS) is a rapidly progressing and invariably lethal neurodegenerative disease caused by the selective death of lower neurons in the spinal cord and upper motor neurons, and resulting in the paralysis of voluntary muscles [1]. The familial and sporadic forms of the disease are clinically indistinguishable and have been proposed to share a common pathogenesis [1]. Mutations in Cu/Zn superoxide dismutase (SOD1) account for approximately 20% of the cases of the inherited form of ALS (FALS) and represent a major known cause of the disease. Transgenic hSOD1^G93A ^mice, which overexpress mutant hSOD1^G93A^, develop the cardinal symptoms of ALS in humans, including muscle paralysis and atrophy [2]. Although the exact molecular mechanisms underlying mutant SOD1 mediated motor neuron degeneration are unclear, the proposed pathophysiological mechanisms of ALS include mitochondrial dysfunction [3], glutamate excitotoxicity [4], disrupted axonal transport [5], and inflammation [6].

Bee venom (BV) is extracted from honeybees and is known as apitoxin. It is used in traditional medicine to reduce inflammation in chronic rheumatoid arthritis and osteoarthritis [7]. Furthermore, we have previously investigated the effects of BV on motor function in a neuroinflammation-related neurodegenerative disease and have demonstrated that BV has anti-neuroinflammatory effects and extends survival in symptomatic hSOD1^G93A ^mice [8].

BV contains a number of bioactive compounds, including histamine, epinephrine, free amino acids, enzymes (e.g., phospholiphase 2; PLA2), and a variety of peptides (e.g., melittin and apamin) [9]. Melittin is a strongly basic, 26-amino acid polypeptide that constitutes 40 to 60% of dry whole honeybee venom, and the peptide has various biological, pharmacological, and toxicological actions, including strong surface activity on cell lipid membranes hemolyzing antibacterial, and antifungal activity [10-12], and antitumor properties [13]. Melittin is also thought to be the major biologically active substance in BV that induces the anti-nociceptive and anti-inflammatory effects observed when BV is applied to an acupoint [9]. In addition, the direct injection of melittin into the rat ventral tegmental area causes sensitization to subsequent cocaine administration, as evidenced by increased cocaine-induced locomotor activity, stereotypy, and dopamine release in the nucleus accumbens [14].

However, the effects of melittin on neuroinflammation are still not clear, and the cellular mechanisms that regulate post-translational modification in hSOD1^G93A ^mice remain to be clarified.

To determine the single bioactive element of BV responsible for the observed anti-neuroinflammatory effects in animal models of ALS, we investigated the effects of melittin in symptomatic hSOD1^G93A ^mice.

Melittin treatment was sufficient to improve motor activity compared to age-matched hSOD1^G93A^ mice, and the peptide inhibited the increased neuroinflammation that is responsible for neuronal death. Furthermore, we found that the post-translational modification of α-synuclein is regulated by melittin treatment and that it is mediated by an increase in Heat Shock Protein70 (HSP70) expression and increased proteasome activity. These findings suggest a potential functional link between melittin and the inhibition of neuroinflammation in an animal model of ALS and could have important implications for the treatment of Parkinson's disease (PD) and ALS.

## Methods

### Animals

hSOD1^G93A ^mice were handled in accordance with the National Institutes of Health guidelines (Bethesda, MD). The protocols were approved by the Institutional Animal Care and Use Committees of the Korea Institute of Oriental Medicine. Hemizygous transgenic B6SJL mice carrying a glycine- to-alanine mutation at the 93^rd ^codon of the cytosolic Cu/Zn superoxide dismutase gene (hSOD1^G93A^) were originally obtained from the Jackson Laboratory (Bar Harbor, ME). Transgenic mice were identified by PCR as described previously [39]. All mice were kept in standard housing with free access to water and standard rodent chow.

### Melittin treatment

Melittin was purchased from Sigma (St. Louis, MO) and diluted with saline. Melittin (0.1 μg/g) was subcutaneously injected bilaterally into 98-day-old male hSOD1^G93A ^transgenic mice (hSOD1^G93A^-MT, *n*= 11) at the Zusanli (ST36) acupoint, which is known to mediate anti-inflammatory effects [40]. The male mice were treated with melittin twice a week. The ST36 point is anatomically located 5 mm below and lateral to the anterior tubercle of the tibia in regard to the human acupoint landmark and a mouse anatomical reference [41]. Age-matched control animals were injected bilaterally and subcutaneously with an equal volume of saline at the ST36 acupoint (hSOD1^G93A^, *n*= 10).

### Behavioral analysis (rotarod test)

Mice for this study were trained for 1 week prior to melittin treatment in order for them to adapt to the apparatus. After training of the hSOD1^G93A ^mice, their basal motor performance was measured with a rotarod apparatus (Ugo, Basil, Italy). Motor coordination was assessed as described previously [8].

### Life span study

For lifespan analysis, 98-day-old male hSOD1^G93A ^mice were divided into two groups: saline-treated hSOD1^G93A ^mice (*n*= 11) and melittin treated hSOD1^G93A ^mice (*n*= 11). We defined "death" that as the day on which the mouse stopped breathing. The significance of the difference in the survival of the treated mice was measured with Kaplan-Meier survival curves using Prism 5.0 software (GraphPad Software, CA, USA) and Sigmaplot 10 software (Systat Software, CA, USA). Values were analyzed by a one-way ANOVA followed by a Dunn's multiple-comparison test.

### Tissue preparation and Immunohistochemistry

hSOD1^G93A ^mice were deeply anesthetized with pentobarbital 18 days after the initiation of the melittin or saline treatment and perfused with phosphate -buffered saline (PBS). The spinal cord and brain were removed and fixed in 4% paraformaldehyde overnight at 4°C, transferred to 30% sucrose in PBS, and then frozen at -70°C. Preparation of the spinal cord and brainstem were performed as described previously [8]. Briefly, the spinal cord and brainstem were embedded in OCT compound cut on a cryostat into coronal slices (40 μm thickness). Selected free-floating sections were treated with 0.6% H_2_O_2 _to inactivate endogenous peroxidases and then incubated: Iba-1 (Wako, Osaka, Japan; 1:5000 dilution) or anti-ubiqutin (DAKO, Glostrup, Denmark; 1:2000 dilution). After incubation with primary antibodies, the sections were washed with PBST (0.3% Tween 20 in PBS) and incubated with a 1:1000 dilution of the secondary antibody. For visualizing, the ABC Elite kit (Vector Laboratories: Burligame, CA, USA) and 3, 3'-diaminobenzidine (DAB)/H_2_O_2 _substrate were used. Immunostained spinal cord and brainstem sections were observed with a light microscope (Olympus, Tokyo, Japan). Immunoreactive cells were counted using an image analysis software package (IMT *i*-solution, NJ, USA).

### Western blot analysis

Eighteen days after the treatment of the melittin or saline treatment, the brainstem and spinal cords were dissected and homogenized in RIPA buffer (50 mM Tris-Cl pH 7.4, 1% NP-40, 0.1% SDS, and 150 mM NaCl) containing a protease inhibitor cocktail (Calbiochem, CA, USA). Homogenized tissues were centrifuged at 14,000 rpm for 20 min at 4°C. The protein concentration was determined using a BCA assay (Pierce, IL, USA). Samples denatured with sodium dodecyl sulfate (SDS) sampling buffer were seperated through SDS-polyacrylamide gel electrophoresis (PAGE) and transferred to a nitrocellulose membrane for western blot. For detection of target proteins, the menbranes were blocked with 5% non-fat milk in TBS and then incubated with the various primary antibodies: anti-tubulin (Abcam, Cambridge, UK), anti-p38 (Cell Signaling Technologies), anti-phospho-p38 (Cell Signaling Technologies), anti-Iba-1 (Wako, Osaka, Japan), anti-TNF-α or anti-ubiquitin (DAKO, Carpinteria, CA). The blots were then probed with peroxidase-conjugated antibodies (Santa Cruz Biotechnology, CA, USA) and visualized by using enhanced chemiluminescence (ECL) reagents (Amersham Pharmacia, NJ, USA). An LAS-3000 image analyzer was used for detecting immunoblotted bands (Fujifilm, Tokyo, Japan).

### Proteasomal activity assay

Whole lysates from the spinal cord and brainstem were prepared with lysis buffer containing 20 mM Tris-HCl, pH 7.5, 150 mM NaCl, 5 mM EDTA, and 0.1% SDS and supplemented with 10 mg/ml of aprotinin, 1 mmol/ml PMSF, and 10 mg/ml of leupeptin. The protein concentration was determined using a BCA protein assay kit (Interchim, Paris, France). Proteasome activity was performed using a 20S Proteasome Activity Assay kit (Chemicon Inc., CA, USA). An AMC fluorogenic standard curve was measured by diluting the reconstituted AMC standard in the concentration range of 0.125 -12.5 μM. Tissue lysates were mixed with a proteasome substrate (Suc-LLVY-AMC) and assay buffer (250 mM HEPES, pH 7.5, 5 mM EDTA, 0.5% NP-40, and 0.01% SDS) in a 96-well fluorometer plate. For positive control assays, 20S proteasome positive control was diluted 1:10 in assay buffer (25 mM HEPES, pH 7.5, 0.5 mM EDTA, 0.05% NP-40, and 0.001% SDS). The reaction mixture was incubated at 37°C for 2 hours, and the fluorescence was read using a 350/450 nm filter set in a fluorometer.

### Statistical analysis

All data were analyzed using GraphPad Prism 5.0 (GraphPad Software, CA, USA) and are presented as the mean ± SEM where inducated. A *t*-test was used to compare the significance of the differences of the immunoblotting and immunohistochemical data between the melittin-treated mice and the age-matched untreated hSOD1^G93A ^mice.

## Results

### Melittin treatment increases motor performance in symptomatic hSOD1^G93A ^transgenic mice

**Figure 1:**
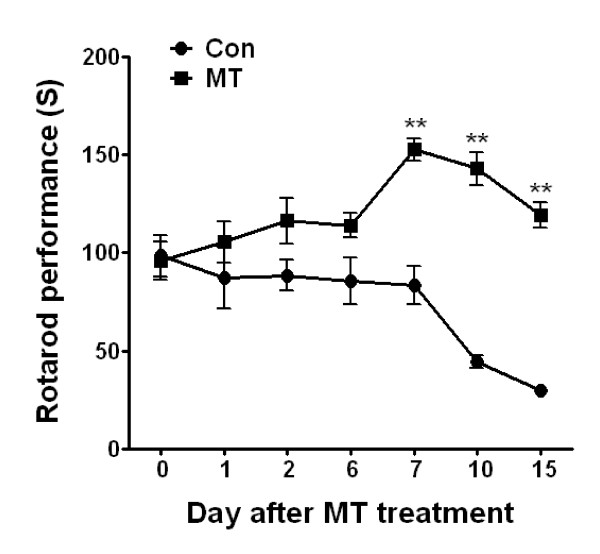
**The effect of melittin treatment on motor activity in symptomatic hSOD1^G93A ^mice**. 14-week-old (98 day old) hSOD1^G93A ^mice were bilaterally treated with saline (*n*= 10/group, circles) or 0.1 μg/ml melittin (*n*= 11/group, squares) subcutaneously at ST36. Based on the results of the rotarod behavioral test for the measurement of motor function, the motor function of melittin-treated hSOD1^G93A ^mice significantly improved compared with saline-treated hSOD1^G93A ^7 days after the initiation of melittin treatment. The values represent as the comparison with melittin-treated hSOD1^G93A ^transgenic mice (**p<0.001). S: second, Con: saline-treated hSOD1^G93A^, MT: melittin-treated hSOD1^G93A^

**Figure 2:**
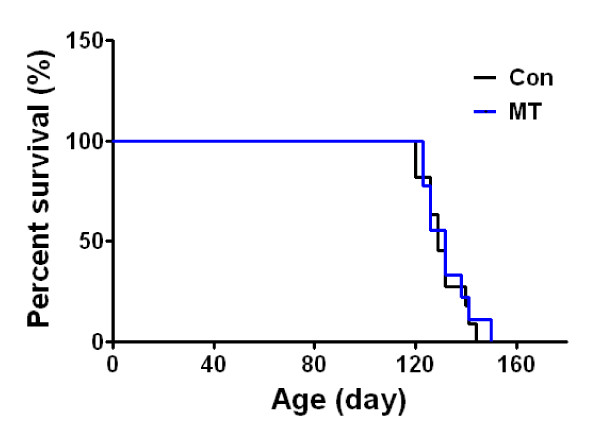
**The effect of melittin on the survival rate of hSOD1^G93A ^mice**. Melittin treatment did not alter the survival of hSOD1^G93A ^mice, as shown by a Kaplan-Meyer analysis. Melittin-treated (0.1 μg/g/98day, n = 11) or saline-treated transgenic hSOD1^G93A ^mice (n = 11) had comparable median lifespans (132 ± 3.2 days and 129 ± 2.5 days, respectively). Con: saline- treated mice, MT: melittin -treated mice

To determine the effects of melittin on the motor function and survival of hSOD1^G93A ^mice, melittin (0.1 μg/g) was subcutaneously injected bilaterally at ST36 (Figure 1). In the rotarod behavioral test, which measures motor function, melittin-treated hSOD1^G93A ^mice (n = 11) displayed a 1.7-fold increase in motor function 7 days after treatment with melittin (Figure 1). Furthermore, melittin-treated hSOD1^G93A ^mice (n = 11) showed a 2.8-fold improvement in motor activity 10 days after melittin treatment compared to age-matched control mice (n = 10) (Figure 1). Disease onset and paralysis, which is a pathological symptom of hOD1^G93A ^mice, was delayed 1 week in melittin-treated mice compared to saline-treated hSOD1^G93A ^mice. Given that melittin treatment improved motor activity and delayed disease onset in hSOD1^G93A ^transgenic mice, we next compared the survival rate using Kaplan-Meier survival analyses. The median survival of the melittin-treated group (132 ± 3.2 days, n = 11) was not significantly different from that of control group (129 ± 2.5 days, n = 11) (Figure 2). It indicates that the melittin treatment does not influence the lifespan of hSOD1^G93A ^mice. However, melittin-treated hSOD1^G93A ^transgenic mice showed delayed disease onset compared to age-matched saline-treated controls (data not shown). Overall, these results indicate that melittin treatment delays the onset of motor dysfunction and disease progression but is not sufficient for increasing the lifespan of hSOD1^G93A ^mice.

### Melittin reduces neuroinflammation in symptomatic hSOD1^G93A ^transgenic mice

**Figure 3:**
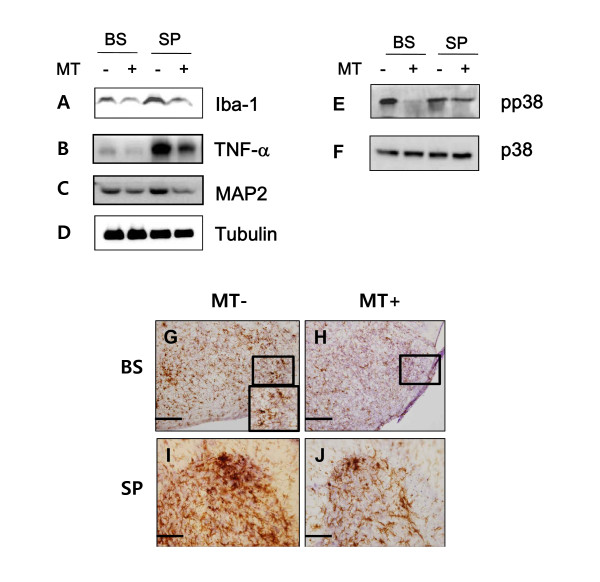
**Western blot and immunohistochemical detection of anti-neuroinflammatory events in the brainstem and spinal cord**. The western blot image is representative of three independent experiments. A representative blot of Iba-1 (A) or TNF-α (B) demeonstrates a significant reduction in the expression of these molecules in the brainstem and spinal cord of melittin-treated hSOD1^G93A ^mice. The expression of MAP2 (a neuron marker) is dramatically increased in both the brainstem and spinal cord of melittin-treated hSOD1^G93A ^mice compared with saline-treated hSOD1^G93A ^mice (C). α-tubulin was used for loading control. The activation of p38 is increased in the brainstem and spinal cord of hSOD1^G93A ^mice, but melittin-treated hSOD1^G93A ^mice showed reduced expression of phospho-p38 (E). The expression of p38 was used as a comparison for the expression of activated phospho-p38. Selected free-floating fifth spinal cord or brainstem section (40 μm) from saline- (*n*= 3) or melittin-treated hSOD1^G93A ^mice (*n*= 4) was stained with an anti-Iba-1. The immunohistochemical study of Iba-1 in the facial nucleus of the brainstem and the anterior horns of the lumbar (L4) spinal cord confirmed that melittin treatment reduced the number of Iba-1-expressing cells in the hSOD1^G93A ^mice compared to age-matched control mice (G-J). The large box presents higher magnification views of the small box in the facial nucleus region of the brainstem in control hSOD1^G93A ^mice (G). Scale bars = 200 μm (G, H). Scale bars = 100 μm (I, J). MT: melittin, BS: brainstem, SP: spinal cord.

Because we have previously demonstrated that treatment with BV treatment reduces microglial activation and neuroinflammation [8], we analyzed the effects of melittin, a component of BV, on microglial activation in symptomatic hSOD1^G93A ^mice. As shown in Figure 3A, Iba-1 expression, which was used as a marker of microglial activation, was significantly decreased in both the brainstem and spinal cord of melittin-treated hSOD1^G93A ^mice. In immuohistochemical study, it confirmed that melittin treatment reduced Iba-1 immunoreactivity largely confined to the facial nucleus of the brain stem and the anterior horn of the lumbar spinal cord (L4) in symptomatic hSOD1^G93A ^mice (Figure 3G - Figure 3J). To further assess the effect of melittin on suppression neuroinflammation by inhibiting of the release of the pro-inflammatory cytokine TNF-α, western blots were performed using an anti-TNF-α antibody The expression of TNF-α was dramatically reduced in the brainstem and spinal cord of melittin-treated hSOD1^G93A ^mice compared to age-matched controls (Figure 3B). Interestingly, melittin treatment increased the expression of the neuronal cell marker MAP2 in both the brainstem and lumbar spinal cord of symptomatic hSOD1^G93A ^mice (Figure 3C).

p38 kinase activity is strongly associated with motor neuron degeneration in this animal model of ALS [15]. Given the previous reports of Yang *et al*. on the effect of BV on p38 activation [8], we examined the effect of melittin on p38 kinase activity in symptomatic hSOD1^G93A ^mice. Phosphorylation of p38 was reduced in the brainstem and spinal cord of hSOD1 transgenic mice following treatment with melittin (Figure 3E). These findings suggest that melittin, a component of BV, has a critical role in inhibiting neuroinflammation in symptomatic hSOD1^G93A ^mice, which results in the attenuation of motor neuron loss.

### Melittin inhibits α-synuclein modification and increases the expression of heat shock protein 70

**Figure 4:**
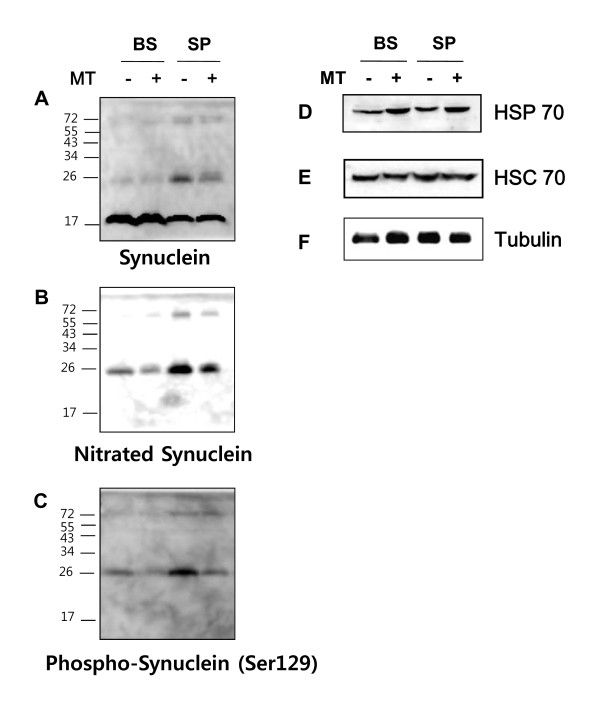
**The Effect of melittin treatment on the post-translation modification of α-synuclein in symptomatic hSOD1^G93A ^mice**. The western blot image is representative of three independent experiments. Higher molecular weight forms of α-synuclein were expressed in spinal cord of hSOD1^G93A ^mice, but this expression was reduced following metlittin treatment (A). The level of nitrated (B) and phosphorylated α-synuclein (C) was decreased in the brainstem and spinal cord of melittin-treated mice. The expression of HSP70 was increased by melittin treatment in the brainstem and spinal cord in hSOD1^G93A ^mice (D). HSC70 (E) and tubulin (F) were used as loading controls.

**Figure 5:**
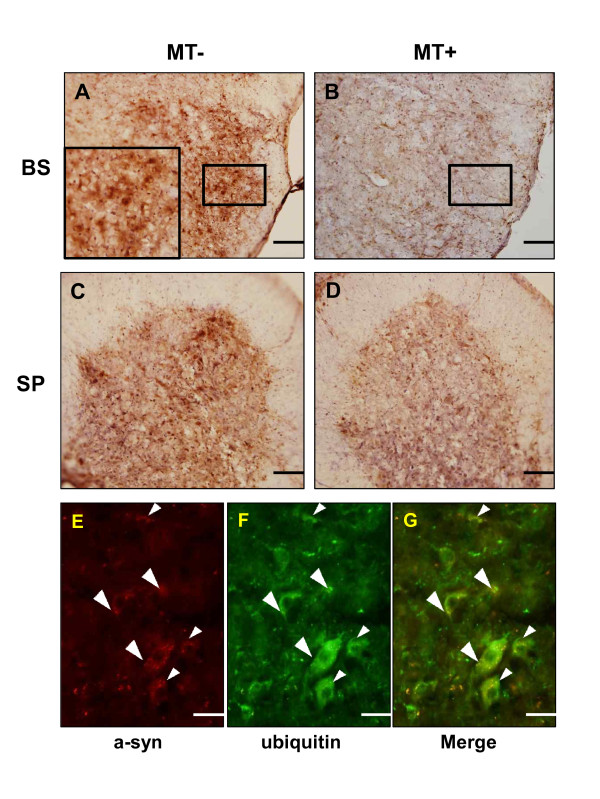
**Co-localization of α-synuclein and ubiquitin in anterior horns of the lumbar (L4) spinal cord in hSOD1^G93A ^mice**. Ubiquitin immunoreactivity (IR) is significantly reduced in the facial nucleus of the brainstem from melittin-treated hSOD1^G93A ^mice (A, B). Scale bar = 200 μm (A, B). In the anterior horn of the spinal cord, the number of ubiquitin-expressing cells was increased in hSOD1^G93A ^mice, but this was reduced by treatment with melittin (C, D). Scale bar = 100 μm (C, D). α-synuclein inclusions (E) were colocalized with ubiquitin (F) in motor neuron of the lumbar (L4) spinal cord in hSOD1^G93A ^mice (G). Arrow head indicates double-labelled cells. Scale bar = 50 μm (E-G). MT: melittin, BS: brainstem, SP: spinal cord.

As a synaptic protein, α-synuclein is a pathological marker for PD and has been implicated in several neurodegenerative diseases, including multiple system atrophy (MSA) [16]. In familial ALS patients, α-synuclein is detected in the glial white matter and glial gray matter [17]. To explore the expression and modification of α-synuclein in symptomatic hSOD1^G93A ^mice, we performed a biochemical study of the brainstem and spinal cord of mice with ALS. Higher molecular weight bands (>17 kDa), indicative modified of α-synuclein were observed in the brainstem and spinal cord of hSOD1^G93A ^mice (Figure 4A). The expression of modified α-synuclein, including Ser129-phosphorylated and nitrated α-synuclein was significantly increased in the brainstem and spinal cord of hSOD1^G93A ^mice (Figure 4B, C). However, melittin-treated hSOD1^G93A ^mice demonstrated reduced expression of post-translationally modified α-synuclein in the brainstem and lumbar spinal cord (Figure 4B, C). To ascertain the mechanism underlying the decreased modification of α-synuclein following melittin treatment of symptomatic hSOD1^G93A ^mice. We examined the expression of HSP 70 in the brainstem and spinal cord of melittin-treated hSOD1^G93A ^mice compared to age-matched controls. As shown in Figure 4D, we observed increased expression of HSP70 in the brainstem and spinal cord of melittin-treated hSOD1^G93A ^mice. Importantly, HSP70 is known to reduce α-synuclein-mediated toxicity and aggregation [18,19]. To determine whether melittin regulates the ubiquitin/proteasome system (UPS) in hSOD1^G93A ^mice, we investigated ubiquitin expression in the brainstem and spinal cord of saline- and melittin-treated mice. As expected, melittin treatment significantly reduced the number of ubiquitin-expressing cells in both the brainstem (Figure 5A, B) and ventral horn of spinal cord in symptomatic hSOD1^G93A ^mice (Figure 5C, D). Furthermore, we confirmed a-synuclein inclusions were colocalized with ubiquitin in motor neurons of ventral horn of spinal cord in symptomatic hSOD1^G93A ^(Figure 5E-5G). These findings suggest that melittin treatment may affect UPS function, which in turn, regulates the post-translational modification (e.g., phosphorylation and ubiquitination) of pathological proteins.

### Reduced proteasome activity is restored in hSOD1^G93A^ mice following treatment with melittin

**Figure 6:**
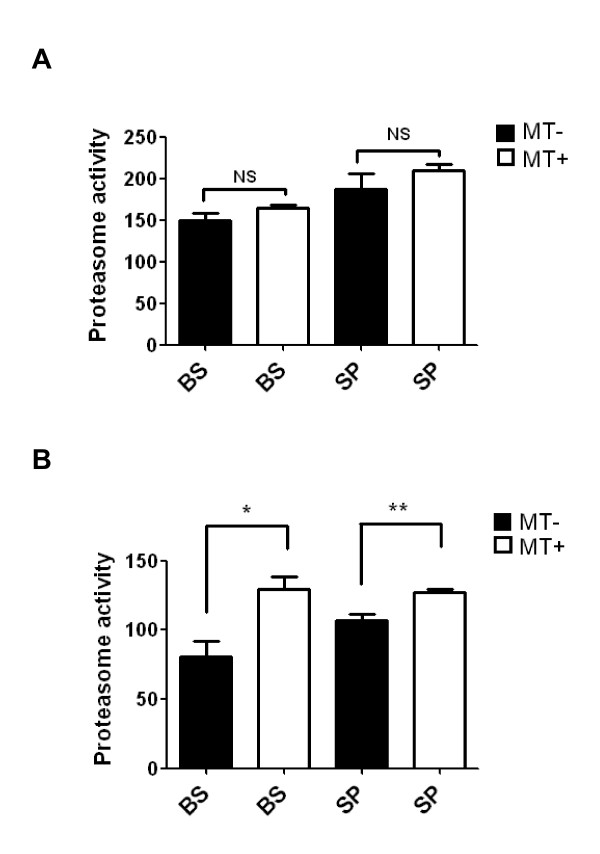
**Melittin treatment restores proteasome activity in the brainstem and spinal cord of symptomatic hSOD1^G93A ^transgenic mice**. Proteasome activity was not changed by melittin treatment in the brainstem and spinal cord of non-transgenic mice (n = 3, A). Melittin treatment was increased proteasome activity in the brainstem and spinal cord of hSOD1^G93A ^mice (n = 3) compared to age-matched hSOD1^G93A ^mice (n = 3, B). NS: no significance, *p<0.05, **p<0.001

To assess whether the observed reduction in total proteasome activity in the brainstem and lumbar spinal cord of hSOD1^G93A^ mice was the result of a decreased number of proteasomes or a reduced proteasomal activity, 20S proteasome activity was examined using a proteasome activity assay with a fluorescent substrate. Total proteasome activity (nmol/min/mg) of the brainstem or lumbar spinal cord in symptomatic hSOD1^G93A ^mice showed each 1.8- fold reduction compared to age-matched non-transgenic mice (Figure 6A, B). In addition, melittin treatment did not significantly affect total proteasome acitivity in non-transgenic mice (Figure 6A). However, proteasome activity following melittin treatment was restored a 50% in the brainstem and a 40% in the lumbar spinal cord of symptomatic hSOD1^G93A ^mice (Figure 6B). These results suggest that reduced proteasome activity results in an increase in the modification of pathological proteins, such as α-synuclein, and that melittin treatment can inhibit the loss of proteasome activity in neurodegenerative diseases.

## Discussion

To improve the treatment of amyotrophic lateral sclerosis (ALS) and to reduce pain and extend the lifespan of afflicted patients, it is important to find molecular markers for the underlying pathological mechanisms of this disease. Pathological mechanisms involved in ALS may include protein misfolding, mitochondrial dysfunction, oxidative damage, glutamate excitotoxicity, and neuroinflammation. We recently demonstrated that bee venom (BV) attenuated neuroinflammation and improved motor performance in a symptomatic animal model of ALS. To determine the critical bioactive compounds of BV critical for anti-neuroinflammation, we studied the effects of melittin, a protein that makes up 40 to 50% of BV, in symptomatic hSOD1^G93A ^mice. We found that administering melittin improved motor activity in these transgenic animals compared with age-matched, untreated animals; melittin administration decreased microglial activity and the expression of the pro-inflammatory factor TNF-α. We also observed that melittin alleviated post-translational modification of α-synuclein and restored proteasome activity in the brainstem and spinal cord of hSOD1^G93A ^transgenic mice at the symptomatic stage.

Neuroinflammation occurs as a result of oxidative and excitotoxic neuronal damage, mitochondrial dysfunction, and protein aggregation [20]. Increased release of pro-inflammatory cytokines, such as TNF-α and IL-6 induces microglia activation in spinal cord of hSOD1^G93A ^mice [21]. Recently, we demonstrated that BV attenuated neuroinflammation due to microglia activation and extended the life span of hSOD1^G93A ^transgenic mice. Consistent with previous reports, the current study demonstrates that melittin, a 26-amino acid polypeptide that is a component of BV, improved motor activity (Figure 1) and reduced neuroinflammatory events in the brainstem and spinal cord of symptomatic hSOD1^G93A ^mice (Figure 3). However, melittin did not extend the survival time of hSOD1^G93A ^mice (Figure 2) even though melittin-treated hSOD1^G93A ^mice delayed disease onset. These findings suggest that anti-neuroinflammation mediated by melittin was not sufficient to prolong lifespan in an ALS animal model.

Neuroinflammation may also be a direct response to protein aggregation [22]. Therefore, it is believed that drugs that modulate inflammation may combat disease progression. The targeting of the different pathogenic events associated with neurodegenerative diseases, such as the clearance of disaggregated proteins, together with neuroprotective and immunomodulatory strategies, may be required for the effective treatment of these diseases. Many diseases are associated with the expression of misfolded proteins that interfere with diverse cellular processes. A number of neurodegenerative disorders, including Parkinson's disease (PD), ALS, Alzheimer's disease (AD), and polyglutamine diseases, are associated with the chronic expression of specific disease-associated proteins, resulting in the accumulation of misfolded species in brain tissue [23]. To protect from the stress of misfolded proteins, all cells express cytoprotective machinery.

PD is the most prevalent neurodegenerative movement disorder and is clinically characterized by rigidity, akinesia, resting tremors, postural instability, and cognitive impairment. PD is considered a synucleinopathy due to the ubiquitous deposition of α-synuclein in the central nervous system of PD patients, with a particularly high enrichment in presynaptic termini, and the associated specific synaptic pathology associated with α-synuclein aggregations [24]. Because α-synuclein is detected in both glial white matter and gray matter in familial ALS patients [17], we investigated the effects of melittin on α-synuclein in hSOD1^G93A ^mice. We observed that α-synuclein phosphorylation and nitration were increased in the brainstem and spinal cord of symptomatic hSOD1^G93A ^transgenic mice and the administration of melittin reduced changes in the post-translation modification of α-synuclein in these tissues (Figure 4A-C). Post-translational modification of α-synuclein at Ser129 has been reported and is the most intensely studied modification of this protein to date. The relatively high abundance of this modified form of α-synuclein in Lewy bodies, and the fact that this post-translational modification occurs at low levels in the absence of large protein aggregates [25], suggests that it may promote aggregation. The phosphorylation of α-synuclein at Ser129 may be a significant pathological event because α-synuclein is not commonly phosphorylated at this site under physiological conditions [26]. Ser129 phosphorylation of α-synuclein was correlated with pathological changes in Aβ and tau *in vivo *[27]. In this study, we demonstrated the co-localization of α-synuclein and ubiquitin in motor neuron of the lumber spinal cord in symptomatic hSOD1^G93A ^transgenic mice (Figure 5). Furthermore, melittin treatment reduced significantly ubiquitinated proteins in the brainstem and the ventral horn of spinal cord in hSOD1^G93A ^(Figure 5). Based on these results and on those other previous studies, we propose that the pathological mechanisms of ALS and PD may be related and, therefore, that melittin treatment may reduce neuroinflammation in PD. However, it should be determined whether melittin directly affects cell toxicity caused PD-related other proteins in the further study.

Various stressful stimuli cause unfolded or misfolded proteins to accumulate within cells. Heat shock proteins (HSPs) recognize misfolded proteins and aid in refolding. In addition to their chaperone activity, HSPs have been shown to facilitate the degradation of highly misfolded proteins by transferring them to the ubiquitin/proteasome degradation system [28]. Gifondorwa *et al*. hypothesized that the administration of HSP70 might delay the progressive loss of motor neurons that has been observed in ALS [29]. HSPs can protect neural cells from various stresses, including oxidative stress and excitotoxicity [30,31]. Furthermore, Batulan *et al*. demonstrated an additional neuroprotective role for the primary stress-inducible HSP70 in an animal model of ALS [32]. Moreover, extracellular HSP70 has been shown to play an important role in immune and inflammatory responses [33]. In this study, we found that melittin administration caused an increased HSP70 expression in the brainstem and spinal cord of symptomatic hSOD1^G93A ^mice compared to the same tissues from age-matched control mice (Figure 4D). These results suggest that melittin regulates the production of misfolded proteins by activating chaperone proteins, including HSP70 and by affecting phosphorylation pathways that could produce pathological proteins, such as α-synuclein and tau in PD and AD.

To determine whether melittin affects protein degradation, we investigated the role of the proteasome in the brainstem and spinal cord of hSOD1^G93A ^mice. Consistent with previous results, we observed an increase in ubiquitinated proteins in the brainstem and spinal cord of symptomatic hSOD1^G93A ^mice (Figure 5A, C). Furthermore, melittin treatment decreased the levels of ubiquitinated proteins in the brainstem and the anterior horn of the spinal cord in hSOD1^G93A ^transgenic mice (Figure 5B, D). The accumulation of misfolded proteins into cellular aggregates is a prominent feature common to most neurodegenerative diseases. These insoluble proteinaceous deposits contain ubiquitin and components of the ubiquitin/proteasome system, including proteins encoded by genes mutated in familial cases (e.g., the ubiquitin ligase parkin/PARK2) [34]. Alternations in the ubiquitin/proteasome system, which are commonly observed in PD, can hinder the degradation of aggregated α-synuclein [35]. In hSOD1^G93A ^mice, the impairment of proteasome function caused by the decreased solubility and aggregation of the mutant SOD1 protein has been demonstrated by Kabahi *et al*., [36]. We observed a 1.8-fold reduction in proteasome activity in the brainstem and spinal cord of symptomatic hSOD1^G93A ^mice compared to age-matched non-transgenic mice (Figure 6A-B). Notably, melittin administration restored a 50% increase in proteasome activity in the brainstem and a 40% increase in the lumbar spinal cord (Figure 6B). Misfolded protein inclusions are a common feature of several neurodegenerative disorders, and their occurrence suggests the function of chaperone protein and ubiquitin/proteasome pathways block to degrade abnormal proteins [37,38].

## Conclusions

The present work demonstrates that melittin reduces neuroinflammatory events and induces a reduction in α-synuclein modification via increased proteasome activity in hSOD1^G93A ^mice, suggesting that proteasome activity and chaperone proteins are responsible for the clearance of the misfolded proteins, such as SOD1 and α-synuclein. The molecular and cellular mechanisms underlying anti-neuroinflammation effects of melittin are not entirely clear and remain to be further clarified by intensive experimental studies. In addition, the link between ALS and PD remains unclear. In further studies, additional experiments are needed to establish whether PD and ALS share common pathological mechanisms and the link between melittin and protein modification remains to be established.

## Competing interests

The authors declare that they have no competing interests.

## Authors' contributions

EJY designed the experiments and analyzed the data as well as wrote the manuscript. SHK executed proteasome acitivity and biochemical study. SCY participated in the tissue processing of animal for all experiments and performed statistical analyses. SML carried out the rota-rod test, immunohistochemistry. SMC discussed with the manuscript. All authors have read and approved the final manuscript.
